# *In Silico* Screening for Novel Inhibitors of DNA Polymerase III Alpha Subunit of *Mycobacterium tuberculosis* (*Mtb*DnaE2, *H_37_R_v_*)

**DOI:** 10.1371/journal.pone.0119760

**Published:** 2015-03-26

**Authors:** Alka Jadaun, Raja Sudhakar D, N. Subbarao, Aparna Dixit

**Affiliations:** 1 School of Biotechnology, Jawaharlal Nehru University, New Delhi, 110067, India; 2 School of Computational and Integrative Sciences, Jawaharlal Nehru University, New Delhi, 110067, India; National Centre for Cell Science, INDIA

## Abstract

Tuberculosis, a pandemic disease is caused by *Mycobacterium tuberculosis* (*Mtb*). DNA polymerase III encoded by *DnaE2* of *Mtb* is specifically required for its survival *in vivo*, and hence can be considered to be a potential drug target. Amino acid sequence analysis of the *Mtb*DnaE2 and its human counterpart does not show any significant similarity. Therefore, a 3D model of the *Mtb*DnaE2 was generated using Modeller 9v10 with the template structure of *E*. *Coli* DNA polymerase III alpha subunit (2HNH_A). The generated models were validated using a number of programmes such as RAMPAGE/PROCHECK, VERIFY_3D, and ProSA. *Mtb*DnaE2 has few conserved residues and four conserved domains similar to that present in DNA polymerase III of *E*. *coli*. *In silico* screening was performed with bioactive anti-tuberculosis compounds and 6-AU (a known inhibitor of DNA polymerase III of *Bacillus subtilis*) and its analogues against the modeled *Mtb*DnaE2 structure. Docking was performed using GOLD v5.2 software which resulted in the identification of top ten compounds with high GOLD fitness scores and binding affinity (X-Score). To further evaluate the efficacy of these compounds, *in silico* ADMET analysis was performed using MedChem Designer v3. Given their high binding affinity to the targeted *Mtb*DnaE2, which is essential for DNA replication in the *Mtb* and good ADMET properties, these compounds are promising candidates for further evaluation and development as anti-tubercular agents.

## Introduction

Tuberculosis, caused by *Mycobacterium tuberculosis (Mtb)*, is a leading cause of death globally. According to the World Health Organisation (WHO), almost 95% of reported cases occur in developing countries and 25% of affected adults die from the infection [1]. The recent emergence of multidrug resistant strains of *M*. *tuberculosis* has further complicated the challenge of curing tuberculosis [2, 3]. Therefore, understanding the molecular mechanisms of the *Mtb* infection can help in the development of new drugs that may be more effective than traditional therapies. Analysing the genome sequence of the *Mtb* and human allows one to identify unique enzymes/proteins that are present only in the pathogen’s metabolic pathway, and not in the host’s [4]. Such unique proteins exclusively present in the pathogen can thus be targeted as potential drug targets [5].

DNA polymerase III (DnaE2) is one such enzyme that barely shares any similarity with the proteins involved in the host’s DNA replication machinery. DnaE2 belongs to the Y family of error prone DNA polymerases that has been reported to be responsible for pathogen survival and drug resistance [6]. Hence, its inactivation would impede *Mtb’s* survival within the host [7, 8]. DNA polymerase III is strongly conserved in a broad group gram-positive pathogens such as *Staphylococcus*, *Streptococcus*, *Enterococcus*, *and Mycoplasma* [9], and has been considered to be a drug target [10]. Many deoxyribonucleotide analogues act as inhibitors or a substrates for DNA polymerase and can inhibit proliferation [11]. An analogue of dGTP, 6-anilino-1H-pyrimidine-2, 4-dione (6-AU) is one of the most common drugs that target DNA polymerase III of gram positive bacteria [12, 13].

In the present study, we have evaluated the therapeutic potential of a large number of compounds against the DNA polymerase III alpha subunit of *Mtb (Mtb*DnaE2). The DNA polymerase III alpha subunit of *M*. *tuberculosis (H*_*37*_ *Rv*) which was selected, did not show any significant sequence identity (BLASTP threshold e value < 0.005) with the human proteins. Since the crystal structure of the *Mtb*DnaE2 has not been determined yet, comparative model of the *Mtb*DnaE2 was generated using DNA polymerase III (2HNH_A) of *E*.*coli (DNA* polIIIα) as the template. The best models were validated by various structure verification programs. Its conserved residues and domains were analyzed in order to predict action mechanisms. *Mtb*DnaE2 model was used for structure based drug designing. After docking, lead compounds that bind to *Mtb*DnaE2 were identified as potential inhibitors of *Mtb*. We have also analyzed the efficacy of top compounds through Adsorption, Distribution, Metabolism, Excretion, and Toxicity (ADMET) studies.

## Materials and Methods

### Sequence analysis

The sequence of *Mtb*DnaE2 (Accession Number NP_217887.2) was obtained from the NCBI database (www.ncbi.nlm.nih.gov) [14]. BlastP was used to identify the suitable template in the Protein Data Bank (PDB) for modeling of the target sequence. The identified top hits included replicative enzymes PDB entries 2HPI_A, 2HNH_A, 3HQA, 3EOD, and 4JOM_A [15–19]. The PDB entries 3HQA and 3EOD were not considered as crystal structures of these templates have only 73 and 130 amino acid (aa) residues respectively. Pairwise amino acid sequence alignment of the selected template and the target sequence was performed using ClustalW for identification of conserved residues in the target [20].

### Comparative modeling and validation, and conserved domains prediction

Though the crystal structure of the E. *coli DNA*polIIIα (PDB entry 2HNH_A), retrieved from the protein data bank (http://www.rcsb.org/pdb/home/home.do) [21] does not have the C- terminal region (911–1160 aa), it was considered to be the best template with 33% sequence identity, based on its higher sequence coverage and resolution (2.30 Ǻ, R value = 0.190,R free 0.258) in comparison to that of other available structures (S1 Table). Models were generated using Modeller 9v10 software [22]. The final model of the *Mtb*DnaE2 was visualized using PyMOL v1.7 viewer [23]. *Mtb*DnaE2 models were validated using verification programs such as RAMPAGE (http://mordred.bioc.cam.ac.uk/~rapper/rampage.php) [24]/ PROCHECK and VERIFY_3D available through NIH (http://nihserver.mbi.ucla.edu/SAVES) [25]. RAMPAGE/PROCHECK provide positional information of each amino acid residues between phi (ϕ) and psi (ψ) angles in a Ramachandran plot [26]. VERIFY_3D is a unique type of verification program that takes into account the location and environment of amino acids and verifies the compatibility of the model from 3D to 1D [27]. ProSA (Protein Structure Analysis) program that analyzes the interaction energy of each amino acid residue in the model, was used to confirm model stability and accuracy on the basis of energy and Z score [28]. Conserved domains were predicted using Pfam server (http://pfam.sanger.ac.uk/)[29].

### *In silico* screening of anti-tuberculosis (bioactive) compounds and, 6-AU and its analogues against the *Mtb*DnaE2

*In silico* screening of anti-tuberculosis (bioactive) compounds was performed against the *Mtb*DnaE2 comparative modeled structure. Bioactive compounds (49413) were obtained from ChEMBL database [30]. A known inhibitor of DNA polymerase III of *Bacillus subtilis*, 6-AU, and its 20 analogues with 95% structural similarity were screened from PubChem Similarity Search [31]. Three dimensional (3D) conformations of all compounds were generated using the CORINA program v3.2 [32].

GOLD v5.2 (Genetic Optimization for Ligand Docking) molecular docking program was used with default parameters for *in silico* screening of all the above compounds against the modeled *Mtb*DnaE2 structure to identify potential inhibitors as it performs flexible docking using genetic algorithm, thus allowing better binding of ligand at a specific site of receptor [33]. The binding pocket residues for *Mtb*DnaE2 model were predicted using pocket finder (http://www.modeling.leeds.ac.uk/pocketfinder) [34]. The predicted residues were cross-checked with the template (2HNH_A). Protein preparation with binding pocket information and ligand library preparation were carried out using the docking wizard of GOLD program. A few independent parameters were applied for fitness function (hydrogen bond energies, atom radii and polariabilities, torsion potentials, etc.), taken from the GOLD parameter file. Best docked complexes were ranked based on their GOLD fitness score and non-bonded interaction analysis. Binding affinity was calculated using X-Score v1.2.1 [35]. Hydrogen bond contacts, lipophilic interactions and non-bonded contacts were calculated using LIGPLOT v.4.5.3 [36]

### Evaluation of ADMET of top compounds

MedChem Designer v.3 was used to evaluate drug permeability (S+logp), distribution (S+logD), topological polar surface area (TPSA), Moriguchi octanol-water partition coefficient (MLogP), molecular weight (Mwt), and the total number of nitrogen and oxygen atoms[37] Such analysis for any molecule provides information about its adsorption, distribution, metabolism, excretion, and toxicity (ADMET). These features can be used to estimate drug efficacy and suitability.

## Results and Discussion

### Sequence analysis

The *Mtb*DnaE2 (Acc No. NP_217887.3) was selected as a target because it shares very little sequence similarity with the human DNA polymerase alpha subunit (Swissprot ID P09884). PSI-BLAST analysis of *Mtb*DnaE2 and human DNA polymerase alpha subunit showed very little sequence similarity (only 20% similarity spanning only 107 amino acid residues regions of the human (877–983) and *Mtb*DnaE2 (371–469 aa). Pfam database showed no similar functional domains between the two. Therefore, the atomic architecture of the binding site of the targeted ligands with *Mtb*DnaE2 is not likely to be present in human proteins, and thus the ligands are likely to be highly specific towards *Mtb*. The *Mtb*DnaE2 was therefore modeled using a suitable template for molecular docking studies and to identify its inhibitors.

Amino acid sequence alignment of the *Mtb*DnaE2 with *E*. *coli* DNApolIIIα showed that few amino acid residues involved in the catalytic reaction of *E*. *coli* DNApolIIIα [16] were also conserved in the *Mtb*DnaE2 (S1 Fig.). The *Mtb*DnaE2 sequence has conserved amino acids residues comprising P-D-X-D-X-D from 380–385, which are also correspondingly present (400–405 aa) in *E*. *coli* DNApolIIIα. Three acidic residues—D381, D383, and D437 of *Mtb*DnaE2, aligned with *E*.*coli* DNApolIIIα sequences (D401, D403 and D457). The two aspartate residues (D401, D403) have been reported to be involved in phosphotransferase activity with two Mg^2+^ ions [38]. The third aspartate amino acid residue plays a major role in the nucleophilic reaction, during the interaction of incoming nucleotides [39]. As observed in *E*. *coli* DNApolIIIα (G363, S364, and K543), equivalent amino acid residues (G344, S345 and K509) were also highly conserved in *Mtb*DnaE2. The residues G363 and S364 formed a binding pocket for the incoming nucleotide while K543 participated in salt bridge creation with the phosphate group of the terminal 3´ base [40]. Amino acid residues R342 and R370 from the palm domain (corresponding equivalent residues R362 and R390 in *E*. *coli* DNApolIIIα) and R666, R667 from the finger domain of *Mtb*DnaE2 (equivalent to R709 and R710 in *E*. *coli DNA*polIIIα) generally interact with dATP [16]. Thus, the analysis revealed the presence of a number of conserved active residues in *Mtb*DnaE2, as present in *E*. *coli* DNApolIIIα. Hence *Mtb*DnaE2 can catalyze the reaction in a similar manner as reported for *E*. *coli* DNApolIIIα.

The amino acid sequences of the three templates (2HPI_A, 2HNH_A and 4JOM_A) showed similar identity (33%) with the *Mtb*DnaE2. While amino acid sequences of all the three templates showed comparable sequence coverage (excluding the C-terminal region in 2HNH_A structure), 2HNH_A structure is solved at a better resolution (2.30 Ǻ, R value = 0.190, R free = 0.258) when compared to 2HPI_A (3.00 Ǻ, R value = 0.225, R free = 0.275) and 4JOM_A (2.9 Ǻ, R value = 0.197, R free = 0.245) and was therefore selected as the template for comparative modeling.

### Comparative modeling of the *Mtb*DnaE2

**Fig 1:**
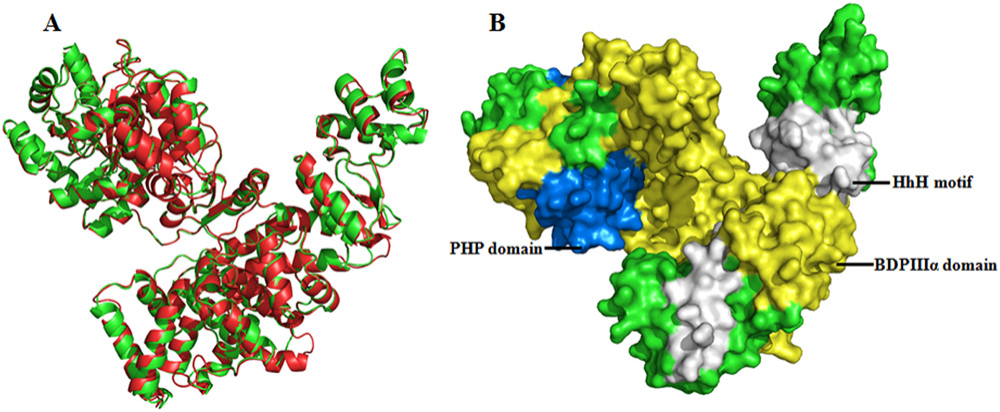
Superimposed Comparative Ribbon Model Structure and Conserved Domains of *Mtb*DnaE2. **(A)** Superimposed comparative model of *Mtb*DnaE2 (green) on the 2HNH_A (red) template with RMSD value (1.4Å). This model was generated by Modeller9v10. The model’s ribbon structure was visualized by PyMoL Viewer **(B)** Connolly surface form of *Mtb*DnaE2 model shows three out of the four conserved domains (identified by domain search)—PHP domain at N terminal, BDPIIIα domain, and HhH motif visualized by PyMOL viewer. The OB fold domain identified in the C-terminal region of *Mtb*DnaE2 can not be seen, due the absence of C-terminal region in the template structure.

For homology modeling, template (2HNH_A) was selected on the basis of resolution,(R = 2.3Å), E-value (6e-134), Max Score (431), Query cover (84%), identity (33%), and positives (50%). The conserved domains analysis also revealed that the template (2HNH_A) has similar domains as in *Mtb*DnaE2 except OB fold (due to the absence of C-terminal region encompassing 911–1160 aa). Based on these analyses, a comparative model with a DOPE score of -96276.38 was generated using the crystal structure of *E coli* DNApolIIIα (2HNH_A) as the template (Fig. 1A). A Ramachandran plot of the best *Mtb*DnaE2 model generated using RAMPAGE showed 91.8% amino acid residues in favorable regions, 7% amino acid residues in allowed regions, and 1.3% amino acid residues in disallowed regions (S2 Fig.). Similar to the template, most of the amino acid residues in the model were arranged in favourable regions that comprised phi (ϕ) and psi (ψ) angles of Ramachandran plot, demonstrating that the modeled *Mtb*DnaE2 structure was reasonably good. Verify-3D showed that 98% of the amino acid residues had an averaged 3D-1D score > 0.2 in the modeled *Mtb*DnaE2 structure (S3A Fig.). Using ProSA, the Z-score of the modeled *Mtb*DnaE2 was determined to be -13.9, which is comparable to that of the template *E*. *coli* DNApolIIIα (-16.19) (S3B Fig.). ProSA analysis revealed that most of the residues in the modeled *Mtb*DnaE2 had negative interaction energy, and only a small number of residues displayed positive interaction energy (S3C Fig.). The modeled *Mtb*DnaE2 structure was superposed onto the template (2HNH_A) and calculated RMSD is 1.40 Ǻ, indicating the model to be of good quality (Fig. 1A). These results clearly suggest that the comparative modeled structure of *Mtb*DnaE2 is of good quality, and can be used for further studies in the absence of experimentally driven structure.

Domain analysis using Pfam database shows that the *Mtb*DnaE2 sequence contains four structurally conserved domains—(i) Polymerase and histidinol phosphatases (PHP) domain at the N-terminal (1–166 aa), which exhibits pyrophosphatase activity that hydrolyzes the pyrophosphate by product of DNA synthesis[41], (ii) bacterial DNA polymerase III alpha subunit domain spanning aa 280–691, involved in pathogen replication system, (iii) Helix-hairpin-helix motif (HhH) located at aa767-856, which interacts with DNA with non-sequence specific manner, and (iv) Oligonucleotide/oligosaccharide binding fold domain (OB-fold) at the C-terminal (aa 934–1005), which interacts with the ssDNA [16]. Three of these domains are also present in the template structure (2HNH_A). The OB-fold domain being part of the C-terminal region is not present in the template as well as in the modeled structure (Fig. 1B).

### *In silico* screening of anti-tuberculosis (bioactive) compounds on the modeled *Mtb*DnaE2

To screen for potential inhibitor(s) of the *Mtb*DnaE2, 49413 compounds (with reported anti-tuberculosis activity on the basis of cellular assays, not directed against the *Mtb*DnaE2) were retrieved from the ChEMBL and docked against the modeled *Mtb*DnaE2 structure. Active residues in the modeled structure identified by pocket finder were cross checked with the template (2HNH_A). The GOLD fitness score for the top ranking eight compounds ranged from 77.62 to 84.62 and the predicted binding energy ranged from -7.90 to -9.71 kcal/mol (calculated using the X-Score) (Table 1). These eight ligands have also been reported to be inhibitors of *Mycobacterium tuberculosis* by both *in vitro* and *in vivo* cell based assays [42–44] and not directed specifically against the *Mtb*DnaE2. High GOLD fitness score and binding affinity to the modeled *Mtb*DnaE2 of these compounds suggest that *Mtb*DnaE2 could be the possible site of action of these compounds (Table 1).

**Table 1:** Molecular Docking Results and Interaction Analysis for Identified putative anti-mycobacterium Inhibitors against *Mtb*DnaE2.

S.No	ChEMBL/ Assay IDs	Compound Name	Hydrogen Bonds	Lipophilic Interactions	Non-bonded Interactions	GOLD Fitness Score	X-Score (kcal/mol)	IC50^Ref^
C1	CHEMBL119236	1-[[ethoxy(tetradecyl)phosphoryl] oxymethyl]-3-phenoxybenzene	1; Tyr 799	8; Glu 361,Arg 362, Arg 403, Ile 407, Cys 409, Gly 425, Ala 428, His 602	29	84.62	-8.86	862.29μM^42^
	SID 103345338							
	AID 38730							
C2	CHEMBL326268	2-(1,3-dioxoisoindol-2-yl)ethoxy-heptylphosphinic acid	4; Cys 402, Arg 403, Asn 405, Lys 605	9; Asp 406, Leu 408, Arg 412, Leu 598, Ser 599, His 602, Leu 618, Ser 792, Leu 796	21	81.13	-8	1.47μM^42^
	SID 104036885							
	AID38730							
C3	CHEMBL325149	1-[[ethoxy(nonyl)phosphoryl]oxymethyl]-3-phenoxybenzene	NIL	13; Arg 403, Ile 407, Leu 408, Cys 409, Arg 412, Thr 427, Ala 428, Val 429, Leu 598, His 602, Ser 792, Ser 795, Leu 796	30	80.22	-9.12	14.83μM^42^
	SID 103346167							
	AID 38730							
C4	CHEMBL175147	1-cyclopropyl-6-fluoro-7-[4-[2-[[5-(5-methyl-2,4-dioxopyrimidin-1-yl)-2,5-dihydrofuran-2-yl]methoxy]-2-oxoethyl]piperazin-1-yl]-4-oxoquinoline-3-carboxylic acid	7; Ile 407, Ser 599, His 602, Lys 605, Asp 606, Ser 795, Tyr 799	9; Cys 402, Asp 406, Gln 410, Arg 412, Ala 428, Tyr 603, Leu 618, Ser 792, Leu 796,	38	79.27	-9.7	6.25μg/ml^43^
	SID 103437119							
	AID 143439							
C5	CHEMBL332399	2-[[ethoxy(nonyl)phosphoryl]oxymethyl]isoindole-1,3-dione	2; Arg 403, His 602	8; Asp 406, Ile 407, Leu 408, Arg 412, Leu 598, Ser 792, Leu 796, Tyr 799	27	78.93	-8.54	87.09μM^42^
	SID 103345226							
	AID 38730							
C6	CHEMBL118269	2-[[ethoxy(tetradecyl)phosphoryl]oxymethyl]isoindole-1,3-dione	1; Tyr 799	11; Arg 403, Leu 408, Arg 412, Gly 425, Ala 428, Glu 457, Gln 460, Leu 598, His 602, Ala 619, Leu 796	31	77.87	-8.52	40.990μM^42^
	SID 103345259							
	AID 38730							
C7	CHEMBL177774	benzyl 2-(6-decylsulfanylpurin-9-yl)acetate	2; His 602, Tyr 799	14; Arg 403, Asp 406, Ile 407, Leu 408, Cys 409, Arg 412, Ala 428, Val 429, Gly 594, Leu 595, Leu 598, Ser 792, Ser 795, Leu 796	34	77.74	-8.59	Not determined^44^
	SID 103438626							
	AID 143119							
C8	CHEMBL418868	2-(1,3-dioxoisoindol-2-yl)ethoxy-hexylphosphinic acid	3; Arg 403, Ile 407, Lys 605	7; Cys 402, Asp 406, Leu 408, Arg 412, Leu 598, His 602, Leu 796	18	77.62	-7.9	4.39μM^42^
	SID 103345288							
	AID 38730							

ChemBL compound ID (CHEMBLID); PubChem Substance accession identifier (SID), Assay ID (AID) for the identified compounds namely C1-C8 are given.

**Fig 2:**
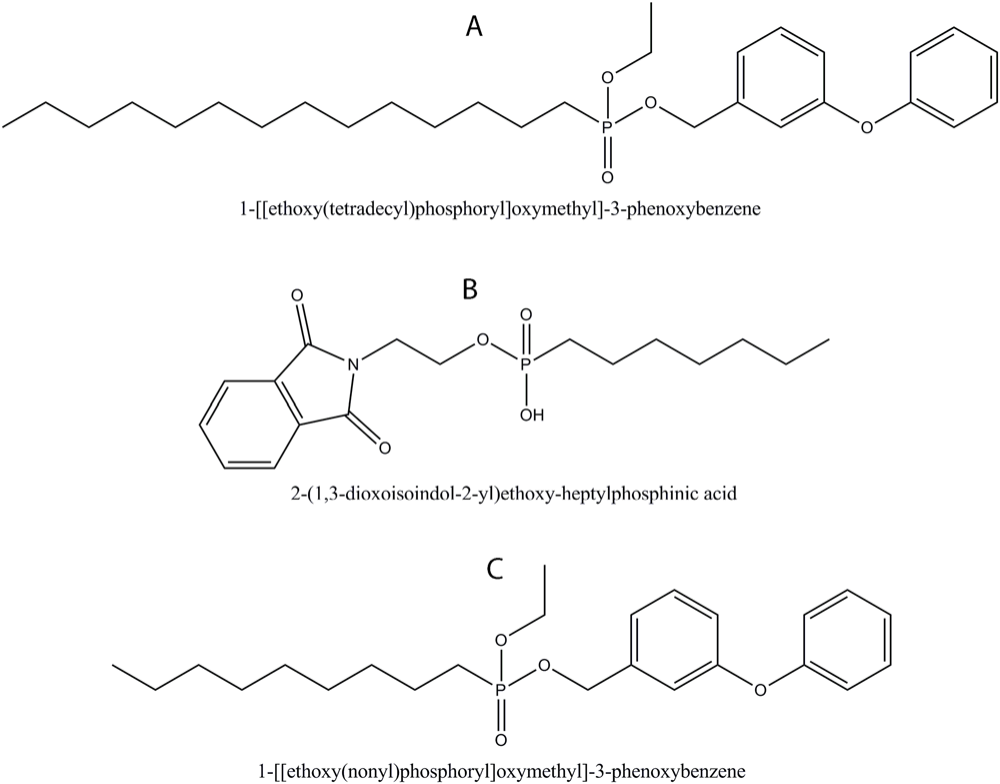
Structure of Top Extracted Compounds by *in silico* Screening. **(A)** 1-[[ethoxy(tetradecyl)phosphoryl]oxymethyl]-3-phenoxybenzene (C1),**(B)** 2-(1,3-dioxoisoindol-2-yl)ethoxy-heptylphosphinic acid (C2), **(C)** 1-[[ethoxy(nonyl)phosphoryl]oxymethyl]-3-phenoxybenzene (C3).

The top compounds (i) 1-[[ethoxy(tetradecyl)phosphoryl]oxymethyl]-3-phenoxybenzene, (ii) 2-(1,3-dioxoisoindol-2-yl)ethoxy-heptylphosphinic acid and (iii) 1-[[ethoxy(nonyl)phosphoryl]oxymethyl]-3-phenoxybenzene, designated as C1, C2, and C3 are shown in Fig. 2A–2C, respectively.

**Fig 3:**
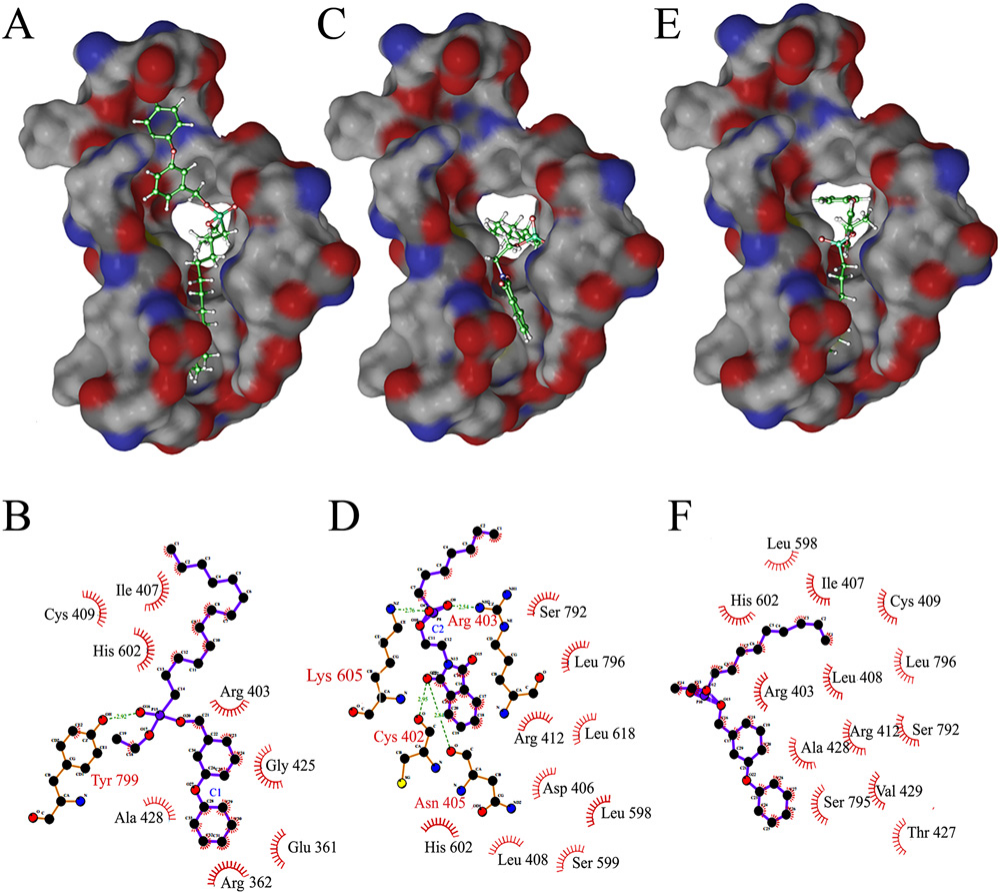
Docked Complex of *Mtb*DnaE2 with ChEMBL Database Compounds. Panels A, C, and E show the electrostatic surface potential of modeled *Mtb*DnaE2 with bound ligands (stick models, shown in atom colors). **(A)** 1-[[ethoxy(tetradecyl)phosphoryl]oxymethyl]-3-phenoxybenzene (C1), **(C)** 2-(1,3-dioxoisoindol-2-yl)ethoxy-heptylphosphinic acid (C2), **(E)** 1-[[ethoxy(nonyl)phosphoryl]oxymethyl]-3-phenoxybenzene (C3) respectively. In ligplot, Panels **B**, **D,** and **F** show 2D representation of ligand C1, C2, and C3 with the interacting amino acid residues of *Mtb*DnaE2. Green dashed lines indicate hydrogen bonds, with the numbers indicating the interatomic distances (Å). The labeled arcs with radial spokes that point toward the ligand atoms show the hydrophobic interactions with the corresponding amino acid residues.

C1 and C2 only have 1 and 4 hydrogen bonding interactions with *Mtb*DnaE2, respectively while C3 did not have any hydrogen bonding interaction with *Mtb*DnaE2. C1 has 8 lipophilic interactions and 29 non-bonded interactions (Fig. 3A &B). Compounds C2 and C3 has 9 and 13 lipophilic interactions and 21and 30 non-bonded interactions, respectively (Fig. 3C-F). High lipophilic interactions of the C3 may accountable for better binding with *Mtb*DnaE2 at -9.12 kcal/mol X-Score (Table 1). Predicted binding energy for C1 and C2 is -8.86 kcal/mol and -8.00 kcal/mol, respectively (Table 1). Residue His602 of *Mtb*DnaE2 were common in top 3 lipophilic interactions, whereas Arg403, Ile 407, Cys409, and Ala428 were common in lipophilic interactions of C1 and C3 with *Mtb*DnaE2.

### Molecular docking of 6-AU and its analogues against *Mtb*DnaE2 comparative model

**Fig 4:**
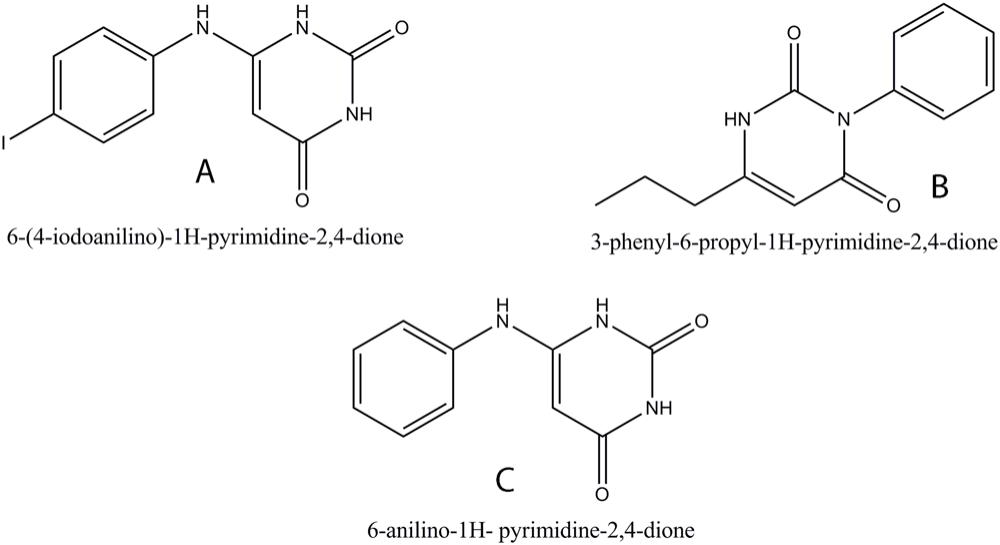
Structure of 6-AU and its Top Analogues **(A)** 6-(4-iodoanilino)-1H-pyrimidine-2,4-dione (C9), **(B)** 3-phenyl-6-propyl-1H-pyrimidine-2,4-dione (C10), **(C)** 6-anilino-1H- pyrimidine-2, 4-dione (6-AU) (control).

6-AU is a known inhibitor of the DNA polymerase III alpha subunit in a number of gram positive bacteria, including *B*. *subtilis* with reported IC50 of 4.7μM [45]. The enzyme interacts with 6-AU compounds through a guanine-like base pairing domain and an enzyme specific aryl domain. The action of these compounds is competitive with dGTP as they are able to form Watson- Crick like hydrogen bonds with an unopposed cytosine residue in the template strand just distal to the DNA primer terminus. The aryl group of these compounds binds near the enzyme’s active site, thus resulting in the formation of an inactive ternary complex [46]. However, 6-AU and its analogues have not been evaluated for their interaction with *Mtb*DnaE2. Therefore, 6-AU and its analogues were identified by a structural similarity search using the PubChem database and were analyzed by docking on the modeled *Mtb*DnaE2 structure. Two analogues of 6-AU—C9 (6-(4-iodoanilino)-1H-pyrimidine-2,4-dione) and C10 (3-phenyl-6-propyl-1H-pyrimidine-2,4-dione, selected on the basis of their GOLD fitness scores (35.81 to 41.56) and X-Score (-7.19 to -7.57 kcal/mol),were analyzed using 6-AU as a positive control (Fig. 4A-C, Table 2). No activity information is available for the identified 6-AU analogues, C9 and C10 [47, 48 **]**. Docking studies revealed that the Arg 403 of the *Mtb*DnaE2 was involved in hydrogen bonding with C10 and 6-AU whereas His 602 hydrogen bonded only with C9 (Table 2).

**Table 2:** Molecular Docking Results and Interaction Analysis for Identified 6-AU and its analogous against *Mtb*DnaE2.

S.No	Compound ID	Compound Name	Hydrogen Bonds	Lipophilic Interactions	Non-bonded Interactions	GOLD Fitness Score	X-Score (kcal/mol)	IC50^Ref^
C9	CID 737913 SID 8052167 AID 372	6-(4-iodoanilino)-1H-pyrimidine-2,4-dione	1; His 602	5; Arg 412, Ala 428, Leu 618, Leu 796, Tyr 799	11	41.56	-7.57	Anti-Mtb activity not reported^47^
C10	CID 22075432 SID 37716788	3-phenyl-6-propyl-1H-pyrimidine-2,4-dione	1; Arg 403	6; Leu 408, Cys 409, Gly 425, Ala 428, Leu 598, Leu 796	20	40.05	-7.44	Anti-Mtb activity not reported^48^
6-AU (Control)	CID 309801 SID 103355885 AID 57364	6-anilino-1H- pyrimidine-2,4-dione	1; Arg 403	3; Arg 412, Ala 428, Ser 792	09	35.81	-7.19	4.7μM^45^

Compound ID (CID); PubChem Substance accession identifier (SID), Assay ID (AID) for the identified compounds are given.

**Fig 5:**
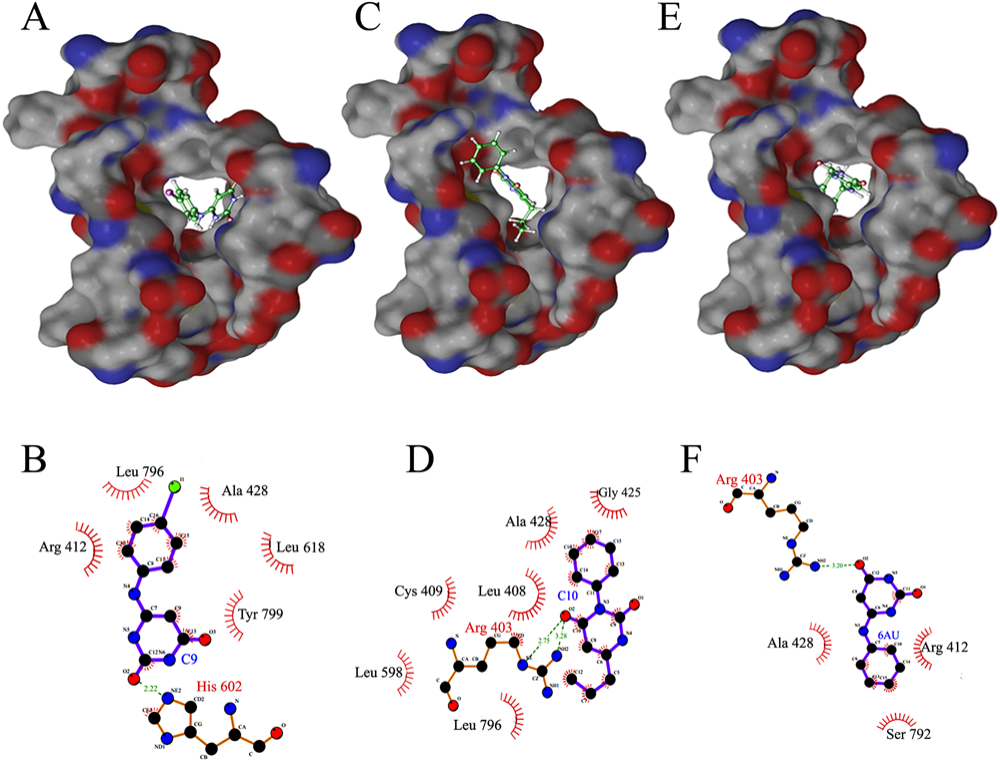
Docked Complex of *Mtb*DnaE2 with 6-AU and its Analogues. Panels A, C, and E show the electrostatic surface potential of modeled *Mtb*DnaE2 with bound ligands (stick models, shown in atom colors). **(A)** 6-(4-iodoanilino)-1H-pyrimidine-2,4-dione (C9),**(C)** 3-phenyl-6-propyl-1H-pyrimidine-2,4-dione (C10), **(E)** 6-anilino-1H- pyrimidine-2,4-dione (control). Panels **B**, **D,** and **F** show 2D representations of ligand C9, C10, and 6-AU with interacting amino acid residues of *Mtb*DnaE2 respectively. Green dashed lines indicate hydrogen bonds, with the numbers indicating the interatomic distances (Å). The labeled arcs with radial spokes that point toward the ligand atoms show the hydrophobic interactions with the corresponding amino acid residues.

The compounds C9 and C10 showed 5 and 6 lipophilic interactions while 6-AU showed 3 lipophilic interactions with the modeled *Mtb*DnaE2 structure (Fig. 5). The compounds C9 and C10 showed 11 and 20 non-bonded interactions whereas the 6-AU showed only 9 non-bonded interactions with the modeled *Mtb*DnaE2 structure (C9, Fig. 5A&B; C10, Fig. 5C &D and 6-AU, Fig. 5E &F). Residue Ala 428 residues of the *Mtb*DnaE2 were commonly involved in lipophilic interactions with all three compounds, whereas Arg 412 showed lipophilic interaction with only C9 and 6-AU (Fig. 5B&F). Ser 792 showed lipophilic interaction only with the 6-AU (Fig. 5F, Table 2). Since these residues (G363, S364, and K543) are involved in nucleotide binding and in salt bridge formation with the phosphate group of the terminal 3′base [16], the selected compounds’ interaction with the equivalent residues in *Mtb*DnaE2 is expected to inhibit its polymerization activity. The binding energy calculated using X-Score of C9 and C10 are -7.57 kcal/mol and -7.44 kcal/mol respectively, which were slightly lower than that determined for 6-AU (-7.19 kcal/mol) (Table 2). This interaction study suggested that two analogues of 6-AU, C9 and C10, showed better GOLD fitness score and binding energy than 6-AU, possibly due to the presence of different functional groups.

### ADMET analysis of the identified compounds

MedChem Designer was used for ADMET analysis of the identified compounds [37]. Generally, lipophilicity is the logarithm value of the partition coefficient P (logP) between octanol and water (buffer), which explains the partition of the unionized (neutral) form of the compound, whereas logD describes the total partition of both the ionized and the unionized forms of the compound. Compounds C1 and C3 identified from ChEMBL showed log p value more than 5 indicating their lipophilic proprieties, whereas compound C2, 6-AU and its analogues C9 and C10 showed low logP scores of 1.59, 0.155, 0.847 and 2.0, respectively, indicating their hydrophilic nature. When compounds are ionized at various pH, their logD value differs from that of logP. Acidic compounds show lower logD value [49]. Thus, similar logP and logD values for compounds C1 and C3 (Table 3) suggest that they exist in their unionized forms (Table 3). Compouinds C2, 6-AU, C9, and C10, were found to have lower logD values in comparision to their logP (Table 3), indicating their acidic nature. MlogP (Moriguchi octanol-water partition coefficient) is well known and is traditionally used in QSAR model structure analysis. It describes the lipophilicity of a compound, which indicates the penetration of the compound from aqueous solutions to lipid-rich zones. Moriguchi's logP (MLogP) of greater than 4.15 suggests that the compound would be poorly absorbed [50]. The calculated MLogP of compounds C1 and C3 were found to be 5.625 and 4.618, respectively suggesting that these compounds are likely to be poorly absorbed. On the other hand, compounds C2 6-AU and its analogues (C9 and C10) showed calculated MLogP value significantly less than 4.15, suggesting that these compounds would be easily absorbed.

**Table 3:** ADMET analysis of top interactive compounds with *Mtb*DnaE2 comparative model.

Compounds name	S+logP	S+logD	MlogP	TPSA	M_NO	MWt
1-[[ethoxy(tetradecyl)phosphoryl]oxymethyl]-3-phenoxybenzene **(C1)**	8.816	8.816	5.625	47.92	4	502.76
2-(1,3-dioxoisoindol-2-yl)ethoxy-heptylphosphinic acid **(C2)**	1.589	1.498	2.304	93.39	6	365.45
1-[[ethoxy(nonyl)phosphoryl]oxymethyl]-3-phenoxybenzene **(C3)**	6.574	6.574	4.618	47.92	4	432.62
1-cyclopropyl-6-fluoro-7-[4-[2-[[5-(5-methyl-2,4-dioxopyrimidin-1-yl)-2,5-dihydrofuran-2-yl]methoxy]-2-oxoethyl]piperazin-1-yl]-4-oxoquinoline-3-carboxylic acid **(C4)**	0.199	-0.421	0.657	164.83	13	617.76
2-[[ethoxy(nonyl)phosphoryl]oxymethyl]isoindole-1,3-dione **(C5)**	3.671	3.665	3.408	82.39	6	407.53
2-[[ethoxy(tetradecyl)phosphoryl]oxymethyl]isoindole-1,3-dione **(C6)**	5.904	5.896	4.484	82.39	6	477.66
benzyl 2-(6-decylsulfanylpurin-9-yl)acetate **(C7)**	4.792	3.881	3.464	68.79	6	456.73
2-(1,3-dioxoisoindol-2-yl)ethoxy-hexylphosphinic acid **(C8)**	1.124	1.011	2.062	93.39	6	351.42
6-(4-iodoanilino)-1H-pyrimidine-2,4-dione **(C9)**	0.847	-0.038	1.793	76.55	5	341.19
3-phenyl-6-propyl-1H-pyrimidine-2,4-dione **(C10)**	2.0	1.372	2.446	55.73	4	242.36
6-anilino-1H- pyrimidine-2,4-dione **(6-AU, as positive control)**	0.155	-0.819	1.22	76.55	5	215.29

S+LogP = Permeability, S+logD = Distribution, MLogP = Moriguchi octanol-water partition coefficient, TPSA = Topological Polar Surface Area, M_NO = total number of nitrogen and oxygen atoms (Ns and Os), Mwt = moleculer weight, C = Compound no.

Topological polar surface area (TPSA, an indicator of the H-bonding potential of the molecule on the receptor) analysis [51] showed that 6-AU and its analogue C9 had a TPSA score of 76.55, which is the second highest and similar to that of compound C2 (93.39) (Table 3). Greater numbers of nitrogen and oxygen atoms are responsible for the formation of more hydrogen bonds. The 6-AU C9, and C2 can form strong and greater numbers of H-bonds with the target due to the presence of a high number of nitrogen and oxygen atoms in these compounds (Table 3). Thus, based on the binding affinity and ADMET properties considered collectively, the present study suggests that compounds C2, 6-AU and its analogues (C9 and C10) were more hydrophilic due to more hydrogen bonding, a high TPSA score, and a logP of <5. The number of nitrogen and oxygen atoms was also high. According to Lipinski’s “rule of five’,” a compound with logP <5 can also be orally administered.

It is of interest to note that of the top three compounds C1,C2 and C3, compound C2 showing the best ADMET properties also has been reported to have minimum IC50 value (1.47 μM) against *M*. *Tuberculosis* in cell based assays. The other compounds, C1, and C3-C8 with good ADMET properties have been evaluated against *Mtb*, however show several fold higher IC50 values when compared to compound C2. Also, compounds C9 and C10 which have not yet been evaluated for anti-*Mycobacterial* activity, these compounds (C1, C3-C10) can be used for designing novel analogues which may show lower IC50 values and thus would be more effective.

## Conclusions

*Mtb*DnaE2 and human DNA polymerase α subunit do not show significant sequence similarity in their primary structure. *Mtb*DnaE2 showed few conserved residues and four conserved domains which were also present in *E*.*coli* DNA polymerase III α subunit. Comparative modeling of the *Mtb*DnaE2 was carried out with crystal structure of DNA polymerase III α subunit of *E*.*coli* (DNApolIIIα, 2HNH_A) as a template using Modeller 9v10. A good quality model was generated and was verified by RAMPAGE, SAVES and ProSA energy plot. *In silico* screening of anti-tuberculosis bioactive compounds (total 49413) resulted in the identification of potential novel inhibitors specific to the target *Mtb*DnaE2. The study resulted in the identification of ten putative inhibitors, including two analogues of 6-anilino-1H-pyrimidine-2, 4-dione (6-AU). Docking interaction analysis identified a few common active residues in *Mtb*DnaE2, also present in the template (2HNH_A), that participated in the biochemical reaction. Based on their strong binding to the *Mtb*DnaE2 and ADMET properties, the shortlisted compounds are likely to inhibit the replicative activity of the *Mtb*DnaE2 and can be evaluated as potential anti-tubercle molecules specific to this target.

## Supporting Information

S1 Table Comparative analysis of identified PDB entries for modeling of *Mtb*DnaE2. (DOC)

S1 Fig Amino acid Sequence alignment of the *Mtb*DnaE2 (NP_217887.2) and 2HNH_A (PDB-ID) using ClustalW. The boxed sequence represents the active residues and more conserved in *Mtb*DnaE2 with respect to 2HNH_A template. The fully conserved residues are represented by (*), strong and weak conservation of amino acids is denoted by (:) and (.), respectively.
(TIF)

S2 Fig The Ramachandran plot of *MtbDnaE2* homology model was generated by RAMPAGE. In plot, Glycine represents in cross form, proline in triangle form and other residue signify in square form, The most of the favorable and allowed residues cover -98.0 and 2.0% expected range in plot with high density.
(TIF)

S3 Fig Evalution of *Mtb*DnaE2 model quality. **A.** VERIFY_3D profile (model compatibility from 3D to 1D form) for modeled *Mtb*DnaE2. Scores over 0.2 indicate a high quality model. **B &C** show ProSA energy plots for the modeled *Mtb*DnaE2 structure. ‘B’ and ‘C’ show overall model quality indicated by Z score and local (knowledge-based energy) quality plots, respectively
(TIF)
