# Correction: *In Silico* Screening for Novel Inhibitors of DNA Polymerase III Alpha Subunit of *Mycobacterium tuberculosis* (M*tb*DnaE2, *H_37_R_v_*)

**DOI:** 10.1371/journal.pone.0128613

**Published:** 2015-05-22

**Authors:** Alka Jadaun, Raja Sudhakar D, N Subbarao, Aparna Dixit

There is an error in the second sentence of the second paragraph within the Introduction. The correct sentence is: DnaE2 belongs to the C family of error prone DNA polymerases that has been reported to be responsible for pathogen survival and drug resistance [6].

There is an error in reference 6. The author names are listed incorrectly. The correct reference is: Boshoff HI, Reed MB, Barry CE 3rd, Mizrahi V. DnaE2 polymerase contributes to in vivo survival and the emergence of drug resistance in Mycobacterium tuberculosis. Cell. 2003; 113:183–193.
