# Supplementary material for: In Silico Screening for Novel Inhibitors of DNA Polymerase III Alpha Subunit of Mycobacterium tuberculosis (MtbDnaE2, H37Rv)
Source: PLoS One. 2015 Mar 26;10(3):e0119760. doi: 10.1371/journal.pone.0119760 (PMC4374717; doi:10.1371/journal.pone.0119760)
Supplement: S1 Table — (DOC) [file pone.0119760.s001.doc]

| **S.No.** | **Template** | **No. of aa residues in crystal structure** | **Resolution**  **(R)** | **R-value** | **R-free** | **Max Score** | **Total Score** | **Query cover** | **E value** | **Percentage identity** |
| --- | --- | --- | --- | --- | --- | --- | --- | --- | --- | --- |
| 1 | 2HNH_A | 910 | 2.3 Å | 0.190 | 0.258 | 431 | 431 | 84% | 6e-134 | 33% |
| 2 | 2HPI_A | 1220 | 3 Å | 0.225 | 0.275 | 482 | 482 | 92% | 3e-150 | 33% |
| 3 | 4JOM_A | 918 | 2.90 Å | 0.197 | 0.245 | 437 | 437 | 84% | 3e-136 | 33% |

**S1_Table:** Comparative analysis of identified PDB entries for modeling of *Mtb*DnaE2.
