# Supplementary figures and images for: In Silico Screening for Novel Inhibitors of DNA Polymerase III Alpha Subunit of Mycobacterium tuberculosis (MtbDnaE2, H37Rv)
Source: PLoS One. 2015 Mar 26;10(3):e0119760. doi: 10.1371/journal.pone.0119760 (PMC4374717; doi:10.1371/journal.pone.0119760)

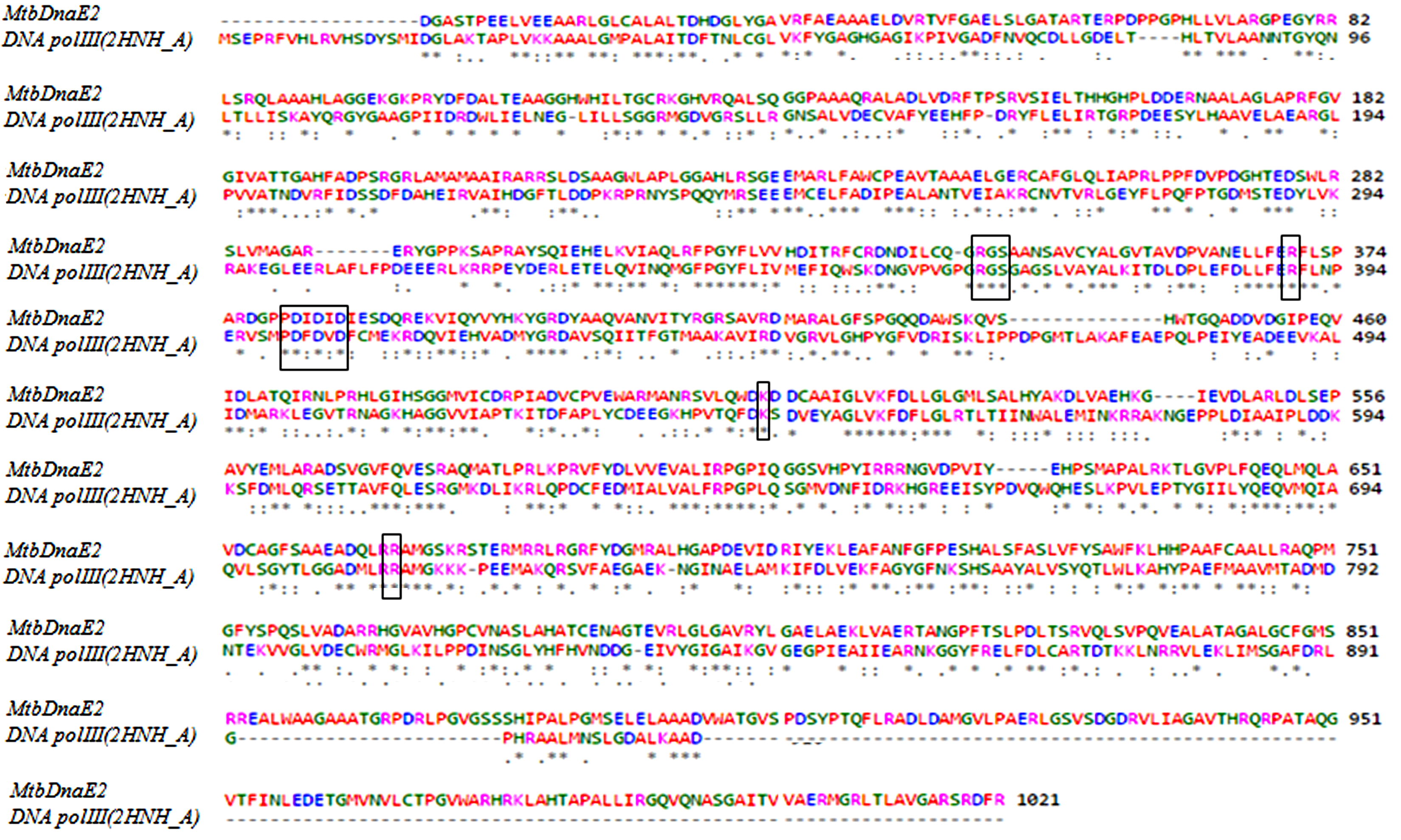

Supplement: S1 Fig — The boxed sequence represents the active residues and more conserved in MtbDnaE2 with respect to 2HNH_A template. The fully conserved residues are represented by (*), strong and weak conservation of amino acids is denoted by (:) and (.), respectively. (TIF) [file pone.0119760.s002.tif]

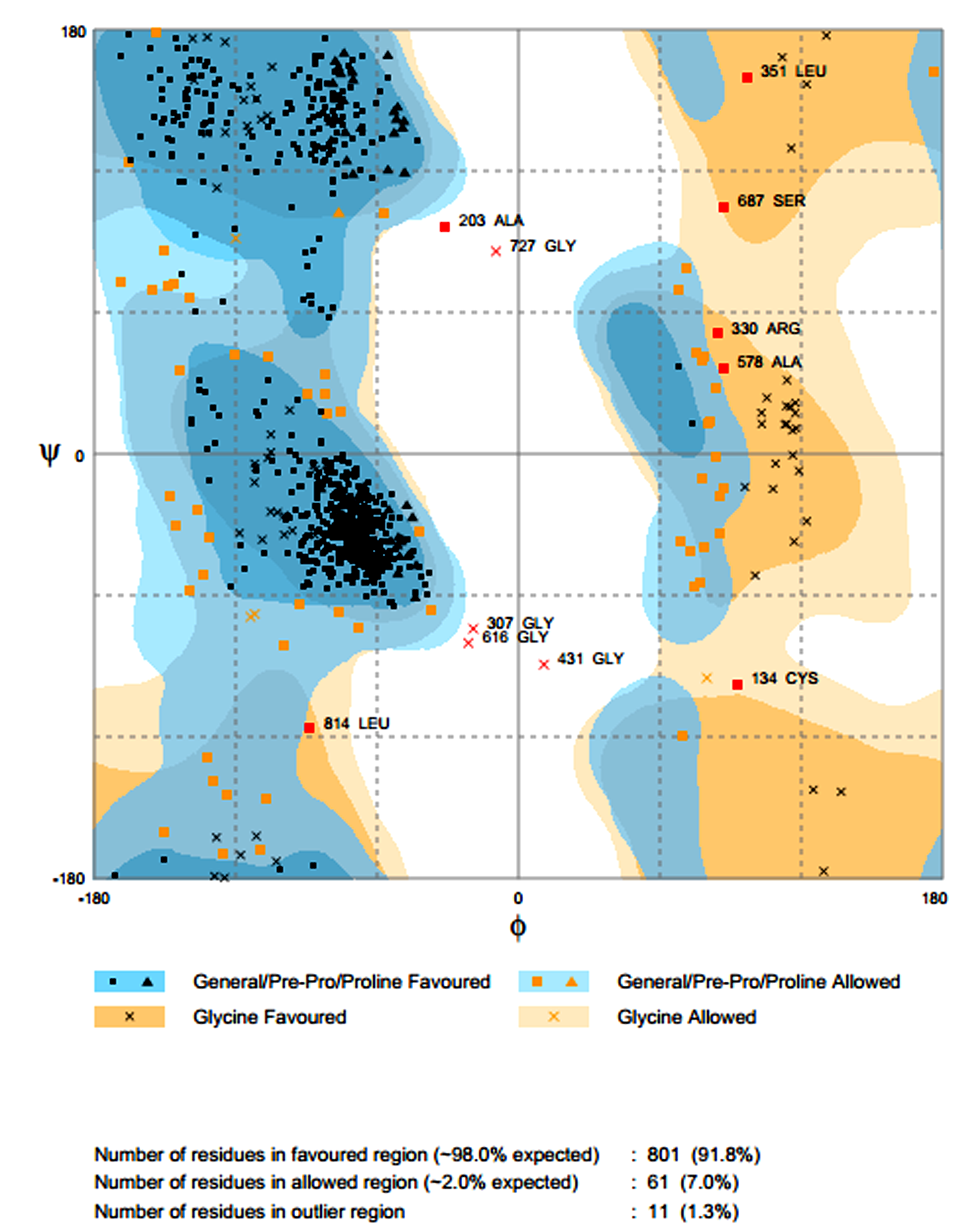

Supplement: S2 Fig — In plot, Glycine represents in cross form, proline in triangle form and other residue signify in square form, The most of the favorable and allowed residues cover -98.0 and 2.0% expected range in plot with high density. (TIF) [file pone.0119760.s003.tif]

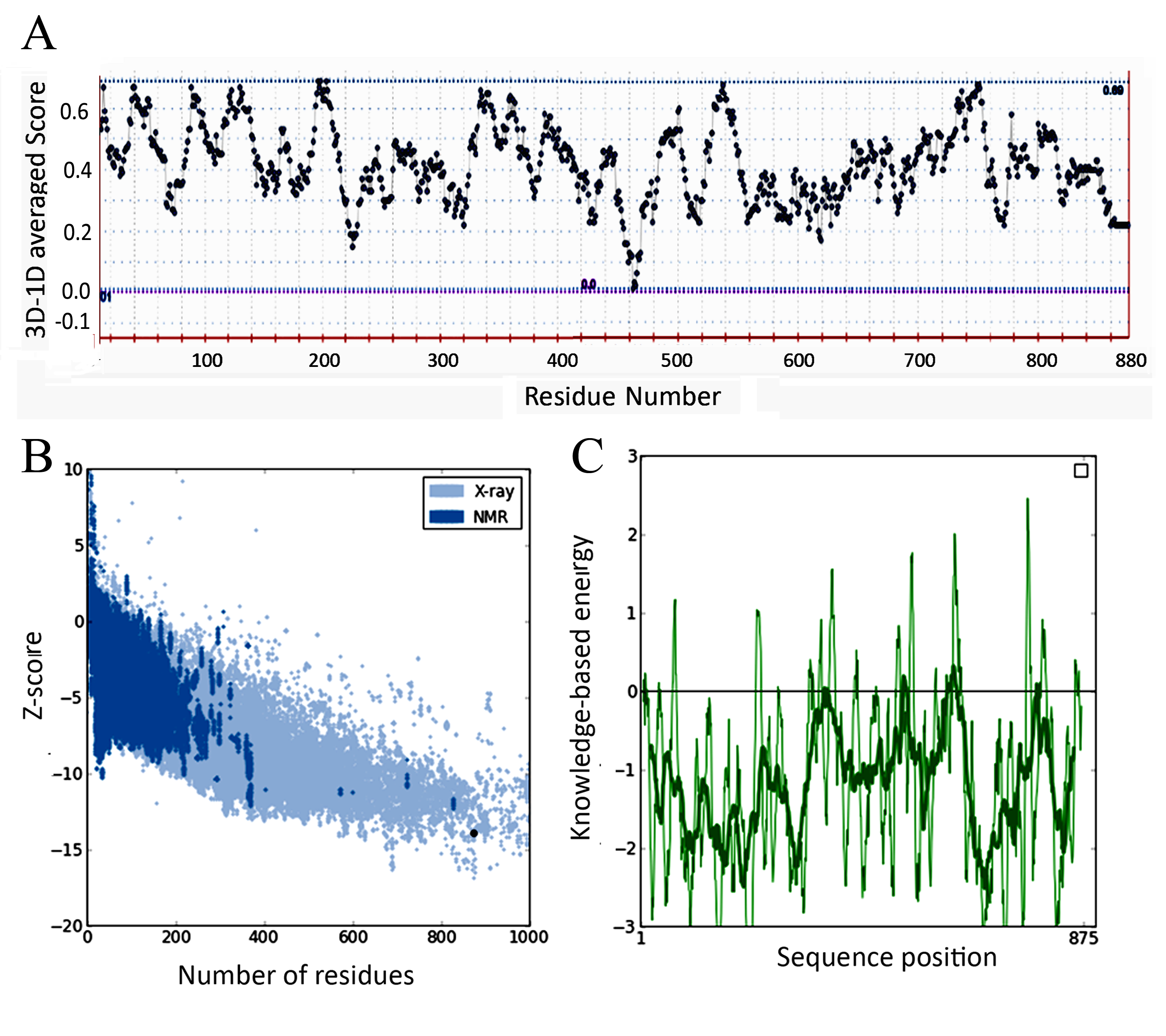

Supplement: S3 Fig — A. VERIFY_3D profile (model compatibility from 3D to 1D form) for modeled MtbDnaE2. Scores over 0.2 indicate a high quality model. B &C show ProSA energy plots for the modeled MtbDnaE2 structure. ‘B’ and ‘C’ show overall model quality indicated by Z score and local (knowledge-based energy) quality plots, respectively (TIF) [file pone.0119760.s004.tif]
